# Meeting the bilingual learning needs of Tibetan minority students in Qinghai Province: A multiple perspective investigation into problems and solutions

**DOI:** 10.3389/fpsyg.2022.937390

**Published:** 2022-10-04

**Authors:** Meng Shi, Jinyan Huang, Guo Lu

**Affiliations:** ^1^The School of Teacher Education, Jiangsu University, Zhenjiang, Jiangsu, China; ^2^Alliance Manchester Business School, The School of Teacher Education, The University of Manchester, Manchester, England, United Kingdom

**Keywords:** Tibetan minority students, Qinghai Province, bilingual learning needs, bilingual education, educational implications

## Abstract

Using student and teacher open-ended questionnaires, and interviews with teachers and school principals and administrators, this study examined the bilingual learning difficulties faced by the Tibetan minority students in Qinghai Province, China, the challenges in meeting their needs, and the suggestions for coping with these challenges. The participants included 200 Tibetan minority students, 20 classroom teachers, and 10 school principals and administrators randomly selected from eight secondary schools located in eight different counties, where there are the most Tibetan minority students in Qinghai Province. The results showed that they experienced considerable difficulties in both spoken and written Chinese, which had prevented them from understanding the lectures, answering questions, interacting with peers in the classroom, and communicating with friends and classmates outside of classroom. The challenges in meeting their bilingual learning needs include: (a) a language choice dilemma for parents, (b) insufficient training of bilingual education teachers, (c) lack of bilingual education resources; (d) short of qualified teachers; (e) lack of parent-school communication; and (f) insufficient training of principals and administrators. Suggestions for coping with these challenges and educational implications are discussed.

## Introduction

Education has always held a special place in protecting the minority students (Wilczynski, 2005; Ringelheim, 2013). Most nations face challenges in educating its ethnically and linguistically diverse students, and the question of how to provide them with equal educational opportunities has been contested in many different contexts (Meusch, 2010; Ringelheim, 2013; Clothey, 2016). Their educational rights include two fundamental dimensions: the right to equal opportunities and the right to transmit their identities through education (Ringelheim, 2013). The bilingual and bicultural education can create equal educational opportunities for the minority or ethnically and linguistically diverse students (Wiese and Garcia, 1998; Meusch, 2010; Movit, 2010; Ringelheim, 2013; Nguyen and Hamid, 2019). The bilingual education study involves language policies and planning, education policies, the sociality of language (i.e., *language* exists in society and they depend on each other and evolve together), family language practice, language teaching, and language learning needs (Clothey, 2005; Baker and Wright, 2016; Buckingham, 2017; Bradbury, 2019).

Bilingual language learning needs may include a wide variety of information about the learner, the teacher, the language being learned, and the context of language learning (Bahador et al., 2014; Brown, 2007). In North America, in order to meet the learning needs of the bilingual students, St. Lambert in Quebec, Canada, took the lead in trying out immersive bilingual education in the 1960s and achieved great success (Dick and Genesee, 2017). In the United States, due to the different attitudes toward immigrants between the federal government and the society, the education of linguistically diverse students becomes a controversial topic (Gandara and Escamilla, 2017; Reiman, 2018; Kappan, 2020). Similarly, in South America, countries like Peru, Ecuador, and Bolivia have opened up new possibilities for indigenous languages and their speakers through bilingual and intercultural education (Hornberger, 2009; Becerra-Lubies et al., 2019). Many countries in Asia including Cambodia, Brunei, India, Malaysia, Philippines, and Vietnam have also experimented with bilingual education (Lee et al., 2015; Buckingham, 2017; Nguyen, 2019). China is a unified multi-ethnic country, and bilingual education of the ethnic minorities^1^ has become an important part of its educational system. Bilingual education for the ethnic minority students, like general education, has its special schools, course curricula, teaching materials, subject teachers, and evaluation system (Ha and Teng, 2001; Ma, 2007; China Education Yearbook [CEY], 2015; Guo, 2019; Wan and Hai, 2019). In the national examinations such as the college entrance examination, tests are available in different ethnic minority languages for the ethnic minority students (Ha and Teng, 2001; China Education Yearbook [CEY], 2015). Chinese higher education also offers special programs for the ethnic minority students (Ha and Teng, 2001; Clothey, 2005; China Education Yearbook [CEY], 2015). Research has started to show that bilingual education in China faces many problems, challenges, and opportunities (Ha and Teng, 2001; Feng, 2007; Ru, 2009; Zhang, 2009; Su and Yuan, 2015; Gao and Wang, 2017). The examination of the challenges in meeting the bilingual learning needs of its minority students would then have important implications.

### An overview of bilingual education of minority students in China

China is a large, united, multi-ethnic nation-state comprised of 56 ethnic groups, with the Han nationality comprising 91.5% and the other 55 ethnic minority groups 8.5% of the total population, respectively (National Bureau of Statistics of China [NBSC], 2010). The number of individuals from the ethnic minority groups exceeds 110 million. They live throughout the country, but about 71.6% of them live in China’s western provinces (Tsung and Cruickshank, 2009; National Bureau of Statistics of China [NBSC], 2010).

Since its founding in 1949, China has stressed the political equality of all 56 ethnic groups and confirmed that it is the basic political right for the minority peoples to use their ethnic minorities’ languages by law. The bilingual education programs for the ethnic minority students were part of the government-led educational campaign. In the minority regions and schools for ethnic minorities, the state carries out educational activities in which the languages of ethnic minorities and commonly used languages of the state are used as the language of instruction for students of ethnic minorities (Ha and Teng, 2001; Ma, 2007).

The purpose of implementing bilingual education is to provide the ethnic minority groups with equal educational opportunities continuously, improve the quality of ethnic minority education, enhance the ability of ethnic minority students to adapt to the social development, and train bilingual and general talents for the country and its ethnic minority regions (Ministry of Education of the People’s Republic of China [MOE], 2012). These programs aim to develop the ethnic minority students’ bilingual competence in the national standard Chinese language (i.e., Putonghua as its spoken form and standard written Chinese as its written form) and their own ethnic languages, hoping that they could be integrated into the mainstream Chinese society and at the same time maintain their own cultural and linguistic integrity and identity (Ha and Teng, 2001; Clothey, 2005; Feng, 2005; Gao and Wang, 2017; Gao and Ren, 2018).

The Chinese government has already established a relatively mature bilingual education system covering the Pre-K to higher education for the ethnic minorities (Ha and Teng, 2001; Guo, 2019). The Chinese central government keeps on stressing the importance of bilingual education of the ethnic minority students, and the local governments continue to provide support to it (Ministry of Education of the People’s Republic of China [MOE], 2012; China Education Yearbook [CEY], 2015). Consequently, the bilingual education of the ethnic minority students has achieved great progress (Ha and Teng, 2001; Ma, 2007; China Education Yearbook [CEY], 2015; Su and Yuan, 2015; Guo, 2019). There are specialized classes, colleges, and universities designed specifically for these students throughout the country, ensuring that they have equal rights to receive education; furthermore, the central government is able to differentiate the various needs of different ethnic minority autonomous regions and provide support accordingly (Ministry of Education of the People’s Republic of China [MOE], 2012; China Education Yearbook [CEY], 2015).

The number of students who can speak both Mandarin Chinese and minority languages has soared after the central government encouraged the bilingual education by adopting the fiscal policy, training experts and teachers in bilingual education, and setting up a large number of primary and secondary schools in the autonomous regions (Ministry of Education of the People’s Republic of China [MOE], 2012; China Education Yearbook [CEY], 2015). According to the education statistics in 2018, the number of teachers who have expertise in bilingual education increased significantly to 206,000. There are 6,521 primary and secondary schools provided the ethnic minority students with bilingual education and over 3,000,000 students nationwide received such education.

However, schools attempting to implement bilingual teaching still face many problems in the areas of curriculum development, course materials, the training of teachers, and the instructional methods and technology (Ma, 2007; Zhang, 2009; Su and Yuan, 2015; Yu, 2015; Zhang and Tsung, 2019). Now the economic and social development in China has brought a shift in government policies and community attitudes towards the bilingual education in the minority regions (Tsung and Cruickshank, 2009).

The proportion of ethnic minorities in Qinghai Province is second only to Tibet and Xinjiang. The Tibetan population is about 1.37 million, accounting for 24.4% of the total population in the province. Nine percent of Qinghai is Tibetan autonomous and Tibetan becomes the major language for communication in the pastoral areas in Qinghai. With the development of the Chinese society, the economic structure of pastoral areas has developed and changed, and the level of urbanization has improved. By 2010, the proportion of Qinghai’s urban population had risen by 15.4% over the past decade. Tibetan people’s demand for Putonghua increases, which has provided the external support for the promotion of bilingual education (Ethnic and Religious Affairs Commission of Qinghai, 2014). Under this change, this study attempted to examine the challenges in meeting the bilingual learning needs of the Tibetan minority students in Qinghai Province and provided suggestions for coping with these challenges.

### Language and bilingual education

Before the review of previous literature on the problems with ethnic minority students’ bilingual and trilingual education in China, it is important to provide a brief summary of the previous literature on language and bilingual education. Specifically, this section discusses the role of language (Tsung and Clarke, 2010; Baker and Wright, 2016; Buckingham, 2017), language learner needs (Richard, 2008; Karimi and Sanavi, 2014; Nguyen and Hamid, 2019), language and bilingual education (Collier and Thomas, 2004; Wu, 2005; Feng, 2007; Baker and Wright, 2016; Gao and Wang, 2017), and students’ home languages and parents’ engagement in developing their proficiency in these languages (Dai, 2004, 2015; Zhang and Yang, 2017; Zhang, 2019; Zhang and Tsung, 2019).

Language both for the individuals and the society is not only an instrument for knowledge and communication, but also a vital tool of cultural development and identity (Ozfidan et al., 2014; Baker and Wright, 2016; Buckingham, 2017). The socially constructed ethnic identity places language at the center of perceived differences between different groups (Tsung and Clarke, 2010; Zhang and Tsung, 2019). Tsung and Clarke (2010) argued that language is an expression of a family’s cultural and ethnic identity, and thus plays a critical role in judging whether one is an ‘insider’ or ‘outsider’. Tsung and Clarke (2010) further confirmed the central role of language as a key symbol of social construction of ethnic identity and a marker of perceived differences among different groups. Therefore, language is not only a means of communication, but also an expression of cultural and national identity.

Learners’ language needs are an umbrella term, which include a wide variety of information such as the learner and the context of language learning (Brown, 2007; Bahador et al., 2014). Richard (2008) argued that learner needs are frequently described in terms of linguistic deficiency; in other words, the difference between a student’s present ability in using the target language and what he or she should be able to do. In order to understand learners’ language needs, we need to perform needs analysis, i.e., gathering information about the learners and the communication tasks for use in syllabus designing before teaching begins (Nunan, 1998; Karimi and Sanavi, 2014).

Bahador et al. (2014) conducted a language needs analysis study among 150 students and suggested that language teachers should adopt different teaching strategies and methods in different grades and schools; at the same time, they should understand the language needs and learning preferences of students and meet their learning needs. The language learning needs of ethnic minority students include both learning needs and social pragmatic needs (Wang, 2013; Nguyen and Hamid, 2019). According to Nguyen and Hamid (2019), individuals’ language choice is influenced by the common values of the group and community to which they belong. In families and communities, older members are key to maintaining the language. Furthermore, the language policy for multilingualism is a concrete safeguard for racial equality and social reduction.

Ngai et al. (2002) affirmed that bilingual education comprises teaching students all topics in two different languages at a school. The purpose of bilingual education is to conserve and develop the linguistic and cultural heritage of the minority groups (Wu, 2005; Baker and Wright, 2016; Gao and Wang, 2017). Culturists believe that preserving a language means preserving a culture that is fading away (Suozzo and Byran, 1996; Cummins, 2001; Ha and Teng, 2001).

Furthermore, for the ethnic minority compatriots, bilingual education enables native speakers to learn a second language to assist their immigrant acculturation to a new community. Bilingual education has also been shown to promote the biculturalism and help to build a student’s self-esteem (Wei and Li, 2010; Li et al., 2018). The important goal of bilingual education is the empowerment of minority students through recognizing their language and culture while improving their self-esteem and keeping the language diversity. Studies since the 1960s have consistently shown that children educated well in two or more languages early in life consistently have an advantage later on (Doughty and Long, 2003; Schluessel, 2007; Nguyen and Hamid, 2019).

Research in the area of language and bilingual education has shown that providing the minority students with bilingual education is the best way to cultivate common cultures and values within a society, and it is also an important means for a country to maintain its cultural diversity in the world (Wan and Xing, 1999; Cummins, 2001; Fang, 2001; Wu, 2005; Feng, 2007; Li et al., 2018). Furthermore, although students’ first language is important for their overall well-being and learning, bilingual instruction has positive effects on their linguistic and educational development (Doughty and Long, 2003; Collier and Thomas, 2004; Goldenberg, 2008; Mcclelland and Sargazi, 2011; Marian et al., 2013; Baker and Wright, 2016). For example, Wu (2005) reviewed China’s bilingual education policies in the past 50 years and stated that bilingual education for ethnic minorities is very necessary in any period. Moreover, minority languages play an important role in strengthening the group identity and social integration. Therefore, China should establish and perfect its bilingual education system for ethnic minorities.

It is important to reemphasize the importance of ethnic minority students’ home languages and parents’ engagement in developing their proficiency in these languages (Dai, 2004, 2015; Zhang and Yang, 2017; Zhang, 2019; Zhang and Tsung, 2019). Their home languages record and carry the history and culture created by the ethnic minorities in the past generations. It is the crystallization of wisdom of ethnic minorities in the past generations and the protection of their languages means the protection of a culture (Dai, 2004, 2015). The culture, history, and customs of the whole nation are behind these languages; and all these things are the carriers of civilization (Zhang, 2019). Furthermore, parents’ support and engagement become important in developing ethnic minority students’ proficiency in different languages (Zhang and Tsung, 2019).

### Problems with ethnic minority students’ bilingual and trilingual education in China

In the past decade, several researchers have examined the problems with ethnic minority students’ bilingual (Zhang and Yang, 2017, 2018; Rehamo and Harrell, 2018; Zhang and Tsung, 2019) and trilingual (Ping, 2016; Feng and Adamson, 2017) education in China. Specifically, these studies specifically examined the Tujia minority students in Hunan Province (Zhang and Yang, 2017), the Uyghur and Kazakh minority students in Xinjiang Uyghur Autonomous Region (Ping, 2016; Zhang and Yang, 2018), the Yi minority students in Sichuan Province (Rehamo and Harrell, 2018), and the Tibetan minority students in Qinghai Province (Zhang and Tsung, 2019). The following section is a review of these studies by the type of educational programs (i.e., bilingual and trilingual) and in chronological sequence.

Many researchers examined the ethnic minority students’ bilingual programs in China (Zhang and Yang, 2017, 2018; Rehamo and Harrell, 2018; Zhang and Tsung, 2019). For example, Zhang and Yang (2017) conducted a field study to examine the current bilingual education situation for the Tujia minority students in Xiangxi Autonomous Prefecture of Hunan Province, China. The participants included two senior teachers, 17 middle school students, two staff from the school office, and two parents selected from three schools in two counties. Both interviews and observations were used for data collection. The results indicated that bilingual education at these three schools face several challenges including lacking teaching resources and task-based or communicative language teaching methods. Furthermore, students and parents have both positive and negative attitudes towards the Tujia language. The sample sizes of the teachers, students, and parents of this study, however, were too small. The results may not be generalized to the bilingual education context for the Tujia minority students in Xiangxi Autonomous Prefecture.

In the following year, Zhang and Yang (2018) examined the medium of instruction policy in the bilingual education of Xinjiang Uyghur Autonomous Region. The participants of this study included seven Uyghur teachers, seven Uyghur students, three Kazakh teachers, six Kazakh students, and three school leaders. Data were obtained from the interviews with the participants and a thorough examination of official documents. The results showed “the discrepancies between national and current regional policies, between planned aim and in-class practice, and between the transitional and balanced types of bilingual education” between the national and regional polices” (Zhang and Yang, 2018, p: 1).

In the same year, Rehamo and Harrell (2018) conducted a large-scale study involving 17 schools and 3,500 students in the upper elementary, middle school, and high school levels, and 350 teachers and administrators. Questionnaires and interviews were used for data collection. The study identified several problems with the bilingual education in Liangshan Yi Autonomous Prefecture of Sichuan Province. For example, inadequate teacher training, outdated textbooks, and students’ insufficient functional literacy in the Nuosu language were the major problems.

Recently, Zhang and Tsung (2019) examined the Tibetan bilingual education policy and family language practice in Qinghai Province between 2013 and 2016. They argued that conflicts exist between the top-down government language policies and bottom-up family language practice. Furthermore, the socio-economic and educational factors outside the scope of government language education policies may have a significant influence on bilingual education practices, multilingual achievement, and the career success of the younger generation of Tibetans. Specifically, the government bilingual education policies can shape family language practice and attitudes towards language. Tibetan parents are in favor of their mother tongue, motivated by family loyalty to the Tibetan culture, identities, and social standing of their Tibetan status in the community. Parents’ language attitude and language use surely affect students’ language learning. They provide a crucial support system for mother-tongue-based bilingual education programs in schools.

In addition, a few researchers examined the ethnic minority students’ trilingual programs in China (Ping, 2016; Feng and Adamson, 2017). For example, For example, Ping (2016) examined the trilingual (i.e., the minority language, L1, Mandarin Chinese, L2, and English, L3) education in Xinjiang Uyghur Autonomous Region. The research argued that it is important to maintain the L1 and its culture because they “contribute to the cultural pluralism and linguistic diversity during the promotion of bilingual and multilingual education” (p: 440).

In the following year, Feng and Adamson (2017) examined the trilingual education (i.e., the home language, Mandarin Chinese and a foreign language) of the ethnic minority students in China. They indicated that most minority students face challenges in learning three languages in schools. Furthermore, they argued that “language policymaking in different domains including families, schools, regions and the state should be informed by research evidence on practical models that are effective in meeting the cognitive and affective needs of children from ethnic minority backgrounds” (p: 1).

The following research gaps were identified. First, previous studies on bilingual learning needs mainly focused on the study of mother tongue and a foreign language (Brown, 2007; Michael, 2011; Karimi and Sanavi, 2014; Shelton-Strong, 2020). Second, the research on the learning needs of ethnic minority students focused more on the learning needs of ethnic minority students of English (Wang, 2013; Li and Zhang, 2015; Li, 2020). Third, ethnic minority students’ bilingual learning needs in China are under researched. Specifically, research on their language choices, attitudes and use of languages at home, and their bilingual learning difficulties is limited. Finally, few studies employed multiple perspectives and triangulated methods in the research designs. This study aimed to bridge these gaps. Specifically, this study used quantitative and qualitative research methods to investigate the bilingual learning difficulties faced by the Tibetan minority students in Qinghai Province from the perspectives of students, parents, teachers, and school principals and administrators. It would provide implications for improving education and language policies as well as promoting educational equity and equal educational opportunities for minority students in China.

### Key terms and expressions

The following key terms and expressions were operationally defined for this study: (a) *Tibetan minority students* specifically refer to the Tibetan students who study in ethnic schools (ethnic junior high schools and ethnic senior high schools) in Qinghai Province and receive bilingual education (Tibetan and Chinese are the medium of instruction), (b) *bilingual education* specifically refers to the education to Tibetan students using both Tibetan and Chinese as the medium of instruction, and (c) *bilingual learning needs* in this study specifically refer to the problems and difficulties Tibetan students encountered in the process of their bilingual learning as well as the expected assistance and advice for improving their bilingual learning outcomes and bilingual competence.

### Research questions

The purpose of this study was to examine the challenges in meeting the bilingual learning needs of the Tibetan minority students in Qinghai Province and provide suggestions for improvement. Specifically, the following four research questions guided this study: (a) what are Tibetan minority students’ specific bilingual learning needs? (b) What are their specific bilingual learning difficulties? (c) What are the challenges in meeting their bilingual learning needs? (d) What are the suggestions for better meeting their bilingual learning needs?

## Materials and methods

### Participants

The participants (see Table 1) of this study included 200 Tibetan minority students, ten school principals and administrators, and 20 classroom teachers randomly selected from eight secondary schools located in eight different counties, where there are the most Tibetan minority students in Qinghai Province. Among the 200 student participants, 90 (45%) were male and 110 (55%) female students; they ranged in age from 13 to 18, and they were 7th to 8th graders in the middle schools, and 10th to 11th graders in the high schools.

**Table 1:** The demographic information of the participants.

	Male		Female	
Group	N	%	N	%
Student	90	45%	110	55%
Teachers	7	35%	13	65%
Principals and administrators	9	90%	1	10%

Total number of students = 200; total number of teachers = 20; total number of principals and administrators = 10.

All 20 classroom teachers were Tibetan. Among them, 7 (35%) were male and 13 (65%) female. They ranged in age from 27 to 48. Four teachers held graduate degrees, two associate degrees, and 14 undergraduate degrees. They had three to 27 years of teaching experience.

Among the 10 school principals and administrators, nine (90%) were Tibetan and one was Han (10%); nine (90%) were male and one (10%) was female. They ranged in age from 36 to 54. Three of them obtained graduate degrees; two associate degrees, and five undergraduate degrees. They had seven to 24 years of teaching experience.

### Instruments

An open-ended student questionnaire was used to collect data from the 200 Tibetan minority students. It collected students’ demographic information and responses to six major questions asking about their specific language and learning needs. The following are a few sample open-ended questions. What language(s) do you speak with your parents and siblings at home? What language(s) do you speak with your friends and classmates at school? What language is important for you to learn and why do you think it is important? What are you major learning tasks at school and outside of school?

In addition, an open-ended teacher questionnaire was used to collect data from the 20 classroom teachers. It consisted of a demographic information section and six major questions about their Tibetan minority students’ specific bilingual learning difficulties. The following are a few sample open-ended questions. What are your Tibetan minority students’ major language difficulties in learning? What are your Tibetan minority students’ major learning difficulties at school?

Furthermore, semi-structured interviews with seven selected classroom teachers and 10 school principals and administrators were conducted between the first author and the participants. The purpose of these interviews was to examine the challenges in meeting Tibetan minority students’ bilingual learning needs and provide suggestions for improvement. The major interview questions were included in Table 2.

**Table 2:** A summary of teacher and principal interview findings.

Main themes	Interview questions	Research questions
*Teacher themes:* (a) a language choice dilemma for parents; (b) insufficient training of bilingual education teachers; and (c) lack of bilingual education resources such as textbooks and other learning materials *Principal themes:* (a) short of qualified teachers; (b) lack of parent-school communication; and (c) insufficient training of principals and administrators	(a) In general, what are the major problems you face in the bilingual education program?	What are the challenges in meeting Tibetan minority students’ bilingual learning needs?
	(b) What are the specific challenges in meeting your Tibetan minority students’ learning needs?	
*Teacher themes:* (a) intensive training of Tibetan minority students’ Chinese proficiency; (b) improving classroom teaching methods; and (c) encouraging parent-teacher communication *Principal themes:* (a) standardizing the Chinese-Tibetan translation of course materials and exams; (b) training qualified teachers for the bilingual education program; and (c) encouraging parent-school communication	(a) What are the priorities for the improvement of the bilingual education program?	What are the suggestions for better meeting Tibetan minority students’ bilingual learning needs?
	(b) What are your specific suggestions for better meeting the Tibetan minority students’ bilingual learning needs?	

### Data collection procedures

The collection of data included three phases which lasted approximately 2 months. Phase One involved the identification of two contact persons who were responsible for distributing printed questionnaires to both teacher and student participants, and then collecting and returning the completed questionnaires to the researchers within the 2 months timeframe.

Phase Two involved the survey data collection. At this phase, the two researchers monitored and checked group returns weekly to see if the answers to a few survey questions changed over time. The purpose of this procedure was to minimize the response bias because one of the main problems with self-report survey research is to what extent the responses accurately reflect the views of the sample and the population (Creswell, 2014; Huang et al., 2021).

Phase Three involved the collection of the interview data from the purposefully selected seven classroom teachers and 10 school principals and administrators representing the 11 participating schools. Among the seven teachers, three were math teachers; three were Tibetan teachers; and one was a Chinese teacher. These interviews were conducted between the first researcher and the participants. They allowed the participants to report their perceived challenges in meeting Tibetan minority students’ bilingual learning needs and their suggestions for improvement.

Although ethics reviews for non-medical research involving human participants are not mandated in Chinese schools (Huang et al., 2021), the researchers provided all student participants and their parents, and teacher and administrator participants with the information of the study. All participants understood that their participation was totally voluntary; only group data would be analyzed and reported; and their responses were strictly confidential.

### Data analysis

The open-ended student and teacher questionnaire data were analyzed both quantitatively and qualitatively. Quantitatively, frequencies and percentages of each recurring theme were calculated. Qualitatively, responses relevant to each research question were sorted and organized by content; conceptually similar responses were grouped together and then categorized according to the recurring themes (Creswell, 2014).

The interview data were analyzed qualitatively. To ensure data integrity and consistency, the data collected were entered into an Excel spreadsheet by the two researchers who have rich experience in qualitative data analysis including coding. Following that, responses under each open-ended interview question and relevant to a specific research question were color-coded and sorted into different categories and subcategories by the two researchers independently first, and then sorted and organized collaboratively based on content; and finally, conceptually similar responses were discussed, grouped together, and then categorized according to the recurring themes. This process was to ensure inter-coder reliability of the qualitative data analysis. To enhance its validity, direct quotes from the participants were also incorporated (Creswell, 2014).

## Results

### Tibetan minority students’ specific bilingual learning needs

The Tibetan minority students’ bilingual learning needs include both language and learning needs. They need both the Tibetan and Chinese languages at school and outside of school. Among the 200 Tibetan students, 179 (89.5%) students reported that they use Tibetan to communicate with their parents and siblings at home, and 21 (10.5%) of them use both Tibetan and Chinese at home; further, 91 (45.5%) students reported that they speak Tibetan with their friends and classmates at school, and 109 (54.5%) of them use both languages to communicate with their friends and classmates at school (see Table 3).

**Table 3:** Tibetan minority students’ language needs.

	Tibetan		Chinese		Both Tibetan and Chinese	
	N	%	N	%	N	%
At school	91	45.5%	/	/	109	54.5%
At home	179	89.5%	21	10.5%	/	/

Total number of students = 200.

However, they had different opinions about the importance of both languages. Among the 200 participants, 14 (10.5%) of them considered Tibetan as an important language for them, 27 (13.5%) of them thought that Chinese is important, and 64 (32%) of them reported that both the Tibetan and Chinese languages are equally important for them. They further stated the following reasons for choosing Tibetan as an important language, “*I am Tibetan*,” “*Most of the people around me speak Tibetan*,” “*It is easy to use*,” “*I just like Tibetan*,” and “*Master Tibetan is the foundation for learning other languages*.” The reasons why they considered Chinese important included “*it is our national language*,” “*I need it to communicate with people around China*,” “*I need it to understand my country and culture*,” and “*it is important for my future education*.”

In addition, 171 (85.5%) of them reported that their parents wanted them to learn both languages well because they need them to succeed at school and in their future careers. According to these participants, since Tibetan is their home language and they can use it at home, their parents always encourage them to speak Chinese with their teachers, classmates, and friends at school.

Finally, English as a third becomes important for them to learn well because it is a required foreign language at Chinese schools. However, its importance was not fully recognized by these Tibetan students and their parents. For example, among the 200 participants, only 28 (14%) of them recognized the importance of English; further, even only eight (4%) of them reported that their parents wanted them to learn English well at school.

In terms of their learning needs, 197 (98.5%) of these Tibetan students reported their learning needs at school and at home. Their learning needs at school included (a) understanding reading materials, (b) understanding class lectures, (c) answering teachers’ questions, (d) interacting with classmates, (e) completing class assignments; and (f) passing quizzes and exams. Their learning needs at home were (a) completing reading assignments, (b) completing written assignments, (c) reviewing class notes and materials, and (d) preparing for quizzes and exams.

It is evident that their language and learning needs are closely related to each other; and in a similar manner, their language proficiencies do impact their learning efficiency and outcomes. Their bilingual learning needs must be met for them to be successful learners.

### Tibetan minority students’ specific bilingual learning difficulties

The Tibetan students’ specific bilingual learning difficulties are reported by their teachers. Among the 20 teachers, 15 (75%) of them reported that their Tibetan minority students had great difficulties in both spoken and written Chinese, and only five of them reported that their students were fluent in both spoken and written Chinese. Students’ lack of Chinese oral proficiency prevented them from understanding the lectures, answering questions, and interacting with peers in the classroom, and communicating with friends and classmates outside of classroom because oral Chinese has become an important part of their bilingual education program, as stated by most of the teacher participants.

Further, 12 (60%) of the teacher participants reported that their Tibetan minority students had considerable challenges in reading course materials, understanding assignment instructions, completing reading and writing assignments, and passing quizzes and exams. They commented that these challenges were caused by their poor reading and writing skills in Chinese; and written Chinese proficiency plays an equally important role in the Tibetan minority students’ bilingual learning process.

Finally, 12 (60%) of the teacher participants reported that their Tibetan minority students’ poor oral and written Chinese caused them substantial difficulties in reviewing class notes and materials, completing homework assignments, and preparing for quizzes and exams when they are at home and far away from their teachers and classmates. Their parents were unable to provide assistance and help because they were usually not highly proficient in Chinese reading and writing.

### Challenges in meeting Tibetan minority students’ bilingual learning needs

The interviews with seven teachers and 10 principals were conducted to find answers to answer the last two research questions, i.e., (a) what are the challenges in meeting Tibetan minority students’ bilingual learning needs? And (b) what are the suggestions for better meeting Tibetan minority students’ bilingual learning needs? Table 2 presents a summary of the findings.

As shown in Table 2, for the challenges in meeting the Tibetan minority students’ bilingual learning needs, the following three major themes were identified from the teacher interviews: (a) a language choice dilemma for parents; (b) insufficient training of bilingual education teachers; and (c) lack of bilingual education resources such as textbooks and other learning materials. Similarly, three major themes were also identified from the principal interviews: (a) short of qualified teachers; (b) lack of parent-school communication; and (c) insufficient training of principals and administrators.

**Teacher themes.** Five out of seven teachers indicated that most of their Tibetan minority students’ parents faced a language choice dilemma between Chinese and Tibetan. “*Some parents think it is better for their children to learn Chinese, but they are not willing to give up Tibetan; there are also some parents who want their children to learn Tibetan, but they fear that their children would have difficulty in finding jobs in the future*” as explained by T4. Further, four out of seven teachers commented that the bilingual education teachers have not received sufficient training. For example, some teachers could not use the appropriate teaching methods to meet their Tibetan students’ learning in the classroom. They were also unable to provide necessary assistance to their students outside of the classroom. In addition, four teachers mentioned that there were not sufficient bilingual education resources such as textbooks and other learning materials. T1 added that “*most of the time, teachers have to translate the materials by themselves, and it is inevitable that they make mistakes in translating materials*.”

**Principal themes.** All 10 principals reported that the shortage of qualified bilingual teachers was the biggest challenge for the bilingual education program. Even the current bilingual education teachers face tremendous challenges in teaching because they did not have the required qualification. For example, P6 commented that “these teachers face great difficulties; they first translate the teaching materials from Chinese into Tibetan, and then prepare their teaching plans and activities.” Five principals identified the lack of parent-school communication as another challenge. “*Parents are usually not active in the education of their children*,” “they [*parents*] *only come to pick up their children*,” and “*they [parents] do not usually communicate with the bilingual teachers and school administrators”* are common responses by these principals. Finally, four principals stated that there was insufficient training of bilingual education principals and administrators. They explained that many principals and administrators did not have the management skills required for bilingual education. “*They [principals and administrators] have not received systematic training although the provincial government has increased funding for principal training in recent years*,” as added by P2.

### Suggestions for better meeting Tibetan minority students’ bilingual learning needs

The following three major themes (see Table 2) were identified from the teacher interviews: (a) intensive training of Tibetan minority students’ Chinese proficiency; (b) improving classroom teaching methods; and (c) encouraging parent-teacher communication. Similarly, three major themes were also identified from the principal interviews: (a) standardizing the Chinese-Tibetan translation of course materials and exams; (b) training qualified teachers for the bilingual education program; and (c) encouraging parent-school communication.

**Teacher themes.** Six out of seven teachers suggested that most of their Tibetan minority students’ Chinese proficiency skills be rapidly increased through intensive training. “*Tibetan students have a relatively poor Chinese foundation; they must have a good foundation in the elementary school, otherwise they will not be able to learn the knowledge well in junior and senior high schools*” as explained by T5. Further, five out of seven teachers commented that the bilingual education teachers adjust their teaching methodology to better serve their students in the classroom. For example, teachers take into consideration their students’ language and learning backgrounds in delivering the lectures. They should also have students centered in the classroom. Additionally, four teachers mentioned that parent-teacher communication be encouraged and promoted. T4 added that “*parents need to be made aware of the importance of their participation of children’s education; and they should contact their children’s teachers on a regular basis*.”

**Principal themes.** Nine out of 10 principals suggested that the Chinese-Tibetan translation of course materials and exams be standardized. P1 made the following comment, “*Chinese-Tibetan translation always causes problems … To meet the bilingual learning needs of the [Tibetan minority] students, it is suggested that the bilingual education course materials and exams be translated by government certified translation agencies and organizations*.” Furthermore, seven principals suggested that bilingual education teachers be trained and re-trained so that they have the right qualifications for this group of Tibetan minority students. Teacher professionalism is also important, as P3 argued, “*teachers with high professionalism are more likely to be accepted by the [Tibetan minority] students and meet their learning needs*.” To encourage parent-school communication was identified as the last suggestion by six principals. They suggested that parents should regularly contact their children’s teachers, school administrators, and even the principals so that they know how their children are doing at school, what they can do to help the school to meet their children’s bilingual learning needs, and how they can help their children learn better.

## Discussion and conclusion

This study examined the challenges in meeting the bilingual learning needs of the Tibetan minority students in Qinghai Province and proposed suggestions for improvement. The first research asked about Tibetan minority students’ specific bilingual learning needs in both areas, i.e., language needs and learning needs. They need to learn Tibetan, Chinese, and English. Most of the students thought Tibetan and Chinese are both important; and their parents want them to learn both languages too. Most of the Tibetans in Qinghai Province live in pastoral areas and grazing is the main mode of production, which determines that they have the Tibetan language needs. Further, they need to learn Chinese in order to integrate into the mainstream society. In schools, Tibetan and Chinese become the languages of communication for students. These findings are consistent with previous research (Feng and Adamson, 2017; Nguyen and Hamid, 2019; Zhang and Tsung, 2019). Nevertheless, they attach less importance to English. Furthermore, they have a wide variety of learning needs at school and at home as well. For example, they need to understand the class lectures at school and complete the homework assignments at home.

The second research question asked about Tibetan minority students’ specific bilingual learning difficulties. They have considerable challenges in Chinese speaking, reading, and writing at schools. They cannot answer questions fluently and accurately in Chinese; they also have challenges in understanding course materials and completing reading and writing homework assignments. Their parents cannot provide them with much guidance and assistance Moreover, they are short of bilingual education resources such as textbooks and other learning materials, and bilingual teachers; and the bilingual instructional methods are relatively backward. These difficulties were also found in the bilingual learning process of other minority students, such as Uyghur and Yi students (Tsung and Clarke, 2010; Zhang and Yang, 2017; Rehamo and Harrell, 2018). In addition, this study found that parents’ lack of communication with their children’s teachers and school administrators becomes another factor that negatively affect students’ bilingual learning. These findings could also inform the bilingual education professionals that students’ parents play an important role in their children’s bilingual education.

The third research question asked about the challenges in meeting Tibetan minority students’ bilingual learning needs. The results from the teacher and principal interviews showed that the short of qualified bilingual education teachers and insufficient training of teachers and school administrators create considerable challenges in meeting Tibetan minority students’ bilingual learning needs. Moreover, the lacks of (a) bilingual education resources, (b) administrators with bilingual educational management expertise, and (c) communication between parents and teachers and school administrators are also the identified challenges. They are the common problems faced by bilingual education programs in many other minority regions in China (Feng, 2007; Ma, 2007; Su and Yuan, 2015; Gao and Wang, 2017; Zhang and Yang, 2017, 2018; Rehamo and Harrell, 2018). These findings may also be informative for helping bilingual teachers of other minority students in China better understand the challenges in meeting the learning needs of their bilingual students.

The final research question was about the specific suggestions for better meeting Tibetan minority students’ bilingual learning needs. The results from the teacher and principal interviews indicated that training students and teachers, improving instructional methods, standardizing course materials, and encouraging parent-teacher and parent-school communications are the suggestions for future improvement. These findings were also consistent with previous research (Ping, 2016; Feng and Adamson, 2017; Zhang and Yang, 2017, 2018; Rehamo and Harrell, 2018).

From the perspective of the teachers, they suggested that the training of teachers be strengthened. They need to be equipped with updated bilingual education knowledge, skills, and instructional methods. Further, students should be provided with a wealth of learning materials. Parents should also be actively involved in students’ learning so that the link between parents and schools can be strengthened. School principals and administrators should play the leading role in training teachers, assisting students, and encouraging parent-teacher and parent-school communication and cooperation. These findings could be important information for both bilingual teachers and school administrators at other ethnic minority schools to improve their programs so that their bilingual students’ learning needs can be better met.

This study has the following two limitations. First, this study used a qualitative approach in its design. Data were obtained from both open-ended questionnaires and interviews. Although the qualitative research approach could provide in-depth understandings the way things are, why they are like that, and how participants perceive them, it has received criticisms (Creswell, 2014). Second, this study selected 20 classroom teachers and 10 school principals and administrators from eight secondary schools located in eight different counties in Qinghai Province as its participants for interviews. These sample sizes were relatively small. These two limitations may limit the interpretation and generalization of the findings of this study to all Tibetan minority students in Qinghai Province and China.

In light of these limitations, the following three conclusions were made. First, it is evident that Tibetan minority students in Qinghai Province face considerable challenges in their bilingual learning at schools. Second, there exist several challenges in meeting these Tibetan minority students’ bilingual learning needs. Finally, although these challenges could create obstacles for the bilingual education programs, there are always ways for improvement.

The results of this study have important implications for Tibetan minority students, their teachers, parents, and school principals and administrators, and the bilingual education policy makers. These findings may also have similar implications for the bilingual education in other provinces across China. First, Tibetan minority students should be aware of their bilingual learning needs and difficulties and find ways to improve their Tibetan and Chinese language proficiencies; furthermore, they should develop specific strategies for their bilingual learning.

Second, the teachers should become aware of their Tibetan minority students’ bilingual learning needs as well as the challenges in meeting their needs. Moreover, it is suggested that they develop their bilingual education competences, including Tibetan and Chinese language proficiencies and bilingual instructional methods and techniques, through professional development opportunities, including external and internal training workshops and seminars.

Third, Tibetan minority students’ parents are encouraged to be actively involved in their children’s bilingual education. They should communicate with the teachers and school administrators on a regular basis in order to understand how their children are learning at schools. Also, they should guide and support their children’s bilingual learning and become part of their bilingual learning.

Fourth, school principals and administrators are suggested to follow the national, provincial, and regional bilingual education polices accurately in their implementation of these policies. Furthermore, they should propose amendments to existing policies in order to better meeting Tibetan minority students’ bilingual learning needs.

Fifth, the bilingual education policy makers should investigate how existing bilingual education policies are implemented in the schools. In addition, they should evaluate these policies and make adjustment regularly so that Tibetan minority students’ bilingual learning needs are properly met.

Finally, the findings of this study may also provide all the above implications for the bilingual education in other provinces across China. Bilingual programs for ethnic minority students in different provinces in China may differ in ethnic minority languages, but their essences are the same. All the bilingual educational programs across the country create equal educational opportunities for the ethnic minority students regardless of their home languages.

## Data availability statement

The original contributions presented in the study are included in the article/supplementary material, further inquiries can be directed to the corresponding author.

## Ethics statement

Ethical review and approval were not required for the study on human participants in accordance with the local legislation and institutional requirements. Written informed consent to participate in this study was provided by the participants’ legal guardian/next of kin.

## Author contributions

MS: conceptualization, literature, methodology, data acquisition, data analysis, and writing—first draft and several rounds of revision. JH: conceptualization, literature, methodology, data analysis, writing—subsequent drafts, reviewing, revising, editing, proofreading, and final draft, preparation and editing for submission. GL: literature, data acquisition support, and data analysis support. All authors contributed to the article and approved the submitted version.

## Conflict of interest

The authors declare that the research was conducted in the absence of any commercial or financial relationships that could be construed as a potential conflict of interest.

## Publisher’s note

All claims expressed in this article are solely those of the authors and do not necessarily represent those of their affiliated organizations, or those of the publisher, the editors and the reviewers. Any product that may be evaluated in this article, or claim that may be made by its manufacturer, is not guaranteed or endorsed by the publisher.

## Footnotes

^1^There are 56 officially recognized ethnic groups in China. Han is the largest group. All the other 55 ethnic groups are called "the ethnic minorities."
